# Targeting Mps1 in combination with paclitaxel inhibits osteosarcoma progression by modulating spindle assembly checkpoint and Akt/mTOR signaling

**DOI:** 10.3892/ol.2021.13058

**Published:** 2021-09-17

**Authors:** Lu Lu, Yuhai Wang, Jian Chen, Ye Li, Qingyang Liang, Feng Li, Chuanchuan Zhen, Kegong Xie

**Affiliations:** 1 Department of Orthopedics, Affiliated Hospital of Youjiang Medical University for Nationalities, Baise, Guangxi Zhuang Autonomous Region 533000, P.R. China; 2 Academy of Orthopedics, People's Hospital of Ningxia Hui Autonomous Region, Yinchuan, Ningxia Hui Autonomous Region 755000, P.R. China; 3 Youjiang Medical University for Nationalities, Baise, Guangxi Zhuang Autonomous Region 533000, P.R. China

**Keywords:** osteosarcoma, paclitaxel, monopolar spindle kinase 1, spindle assembly checkpoint, Akt/mTOR signaling

## Abstract

Osteosarcoma (OS) is the most common malignant bone tumor in children and adolescents and is characterized by early metastasis and frequent recurrence, which greatly affects patient prognosis and survival rates. However, the treatment of OS, its recurrence and subsequent metastasis is now at a clinical bottleneck. To explore new OS chemotherapeutic targets, investigate new therapeutic strategies and improve patient prognosis and survival rates, the roles of paclitaxel (PTX) and monopolar spindle kinase 1 (Mps1) in OS were investigated using *in vivo* and *in vitro* models. Mps1 expression was upregulated in OS samples and associated with patient survival times. Moreover, spindle assembly checkpoint (SAC) activation and upregulation of Akt/mTOR signaling were both positively associated with OS progression. PTX treatment significantly inhibited Mps1 expression, as well as migration of OS cells both *in vitro*. In addition, the combination of Mps1 knockdown and PTX treatment inhibited OS progression *in vivo*. Mps1 overexpression inhibited the expression of SAC markers and upregulated Akt and mTOR expression, while Mps1 knockdown had the opposite effect. Cells subjected to combined Mps1 knockdown and PTX treatment exhibited activation of SAC and inhibition of Akt/mTOR signaling compared with Mps1 knockdown or PTX treatment alone. Based on these observations, Mps1 inhibition combined with PTX treatment may represent a potentially effective strategy for the treatment of OS.

## Introduction

Osteosarcoma (OS) is the most common primary malignant bone tumor, occurring primarily during childhood and adolescence. It is characterized by cell invasion and an early and high metastasis rate (1). Despite decades of OS research, surgery and chemotherapy are still the main treatment strategies for OS; the 5-year overall survival rate for OS patients is 70–80% (2). Moreover, the long-term survival rate is still low amongst patients with OS, due to the high frequency of metastasis and recurrence. Indeed, the 5-year recurrence survival rate is only 17.7% and is associated with age at diagnosis, disease severity, recurrence site and the time of recurrence (3). Therefore, OS treatment has entered a clinical bottleneck, with further breakthroughs required to improve patient prognosis (4). Thus, OS pathogenesis must be comprehensively unraveled, with a concomitant strategy towards the development of new therapeutic drugs.

In the 1960's, paclitaxel (PTX) was identified from the common Pacific Yew (*Taxus chinensis*). With its cytotoxic properties, the molecule attracted attention from researchers, who rapidly discovered its cytotoxic properties could be used for the development of new anticancer drugs (5). PTX promotes microtubule polymerization and stabilization in cells, where it regulates the spindle in mitosis, antagonizes the abnormal division of chromosomes and halts cells in the metaphase stage of mitosis, so as to reduce excessive proliferation and division of anticancer cells (6,7). Monopolar spindle kinase 1 (Mps1) was identified in 1991 in a yeast genetic screen of genes involved in spindle pole replication (8) and plays a central role in ensuring error-free chromosome segregation (9). Mps1 is critical for the recruitment of spindle assembly checkpoint (SAC) proteins to unattached centromeres, formation of the mitotic checkpoint complex (MCC) formation and inhibition of the multi-subunit E3 ubiquitin ligase (APC/C) (10). Inhibiting Mps1 activity leads to premature cell separation from mitosis, resulting in severe chromosome mismatching, non-integer ploidy and eventually cell death (11). The inhibition of Mps1 using specific inhibitors reduces SAC signaling inhibition and returns cells to normal mitotic processes, underscoring its feasibility as a potential new anticancer strategy (11). Currently, Mps1 inhibitors are used in basic research, with excellent anti-cancer effects (12–14). Equally, the synergistic effects of Mps1 inhibitors combined with PTX has been shown to promote tumor cell death by enhancing cell division errors (15,16).

The SAC is the primary cell cycle control mechanism during mitosis, preventing chromosome mis-sets, and regulating the attachment of all sister chromatids to microtubules radiating from the poles of the spindle, thereby inhibiting cell entry into anaphase (17). The PTX regulation of cell mitosis appears to be inextricably linked to SAC regulation (18,19). A previous study showed that taxanes, as microtubule regulators, play apoptotic roles via SAC (20). Therefore, it is important to clarify the relationship between PTX and SAC and determine whether SAC regulation is mediated by Mps1.

The AKT/mTOR signaling pathway plays an important role in the regulation of normal cell growth, metabolism and survival. Activation or mutation of proteins involved in this pathway in human cells commonly occurs in different cancer types and causes a series of cell overgrowth syndrome with potential transit risk (21,22). In recent studies, the role of the AKT/mTOR pathway in OS regulation has been documented (23–25). AKT/mTOR signaling pathway has also been used to investigate the synergistic use of PTX in anticancer mechanisms and confirm that the regulation of AKT/mTOR pathway is expected to address the issues with PTX anti-tumor resistance (26,27). However, whether PTX inhibits OS cell migration and invasion through the AKT/mTOR pathway remains unclear. In the present study, *in vivo* and *in vitro* experiments were performed to demonstrate the role and mechanism of PTX and Mps1 with regard to OS.

## Materials and methods

### 

#### Sample and clinical data collection

Between January 2009 and August 2019, 100 intraoperative tissue specimens from 50 patients undergoing OS surgery in the Department of Pathology, Affiliated Hospital of Youjiang Medical University for Nationalities (Baise, China). These included 50 OS samples and 50 normal tissue samples adjacent to the OS samples. The normal tissue in this study refers to the normal bone tissue adjacent to the enlarged resection of the tumor with a distance >5 cm from the tumor edge during surgical resection of osteosarcoma. Patients (28 male and 22 female) were aged between 11 and 80 years, with an average age of 51±5 years. Preoperative National nosocomial infections surveillance system grade (28,29) was observed in five patients, and grade 0 in 45 patients. All study patients were newly diagnosed OS patients, who had received no other treatment before surgery. Postoperative pathological examination results indicated OS, with no other systematic history of malignant tumors. Inclusion criteria were as follows: i) Patients undergoing osteosarcoma surgery admitted to the Affiliated Hospital of Youjiang Medical University for Nationalities from January 2009 to August 2019 were selected and osteosarcoma specimens were collected; ii) patients have a clear pathological diagnosis; iii) patients with primary osteosarcoma; iv) patients were between 10 and 80 years old; v) patients included both men and women; vi) all patients received the same standard treatment regimen for osteosarcoma; and vii) the clinical cases and follow-up data of the patients were complete. The exclusion criteria were: i) Patients who had been treated with high-dose chemotherapy and other anti-tumor drugs before specimen collection; ii) cases complicated with other malignant tumors; iii) patients with severe heart disease; iv) liver and kidney failure before treatment; and v) perioperative death. A log-rank test was used to analyze Mps1 expression and survival rates of 50 clinical patients with OS data. Prior to sample collection, the study was approved by the Medical Ethics Committee of the Affiliated Hospital of Youjiang Medical University for Nationalities (approval no. YYFy-LL-2018-003). All patients or their parents/legal guardians (for patients <18 years old) provided written informed consent and agreed to the use of their samples in this study.

#### Reverse transcription-quantitative PCR (RT-qPCR)

Total RNA from adjacent normal and tumor tissue from patients with OS was isolated using TRIzol^®^ (Invitrogen; Thermo Fisher Scientific, Inc.) according to manufacturer's instructions. Total RNA was then reverse transcribed to cDNA using the PrimeScript™ RT-PCR kit (Takara Bio, Inc.). The RT reaction conditions were 25°C for 10 min, 42°C for 30 min and 85°C for 5 min. Subsequently, qPCR was performed using SYBR-Green reagent (Yeasen Biotechnology Shanghai) using the following primers: i) Mps1 forward, 5′-CGCAGCTTTCTGTAGAAATGGA-3′ and reverse, 5′-GAGCATCACTTAGCGGAACAC−3′; and ii) GAPDH forward, 5′-CATGTACGTTGCTATCCAGGC−3′ and reverse, 5′-CTCCTTAATGTCACGCACGAT−3′. The GenBank accession number for Mps1 is NM_003318. PCR thermocycling conditions were as follows: 95°C for 2 min, 94°C for 15 sec, 60°C for 15 sec and 72°C for 20 sec, for a total of 40 cycles. Comparative quantification was assessed using the 2^−ΔΔCq^ method (30), using GAPDH as the endogenous control.

#### Histopathology and immunohistochemistry (IHC)

OS and normal adjacent tissue samples were collected during intraoperative surgical resection. Specimens were fixed in 10% formalin for 24 h at room temperature and embedded in paraffin, and were subsequently sectioned into 4-µm sections. Hematoxylin and eosin staining was performed as follows: The slides were incubated with hematoxylin solution in a staining jar for 10 min to stain the nuclei, and then were transferred to a staining jar with eosin solution for 3 min at room temperature. For IHC analysis, deparaffinized sections were pretreated with 0.4% pepsin for 60 min at 37°C, and endogenous peroxidase activity was blocked by treatment with 3% H_2_O_2_ for 10 min at 37°C and incubated with 50 µl of normal 5–10% goat serum blocking antigen for 15 min at room temperature. The following primary antibodies were used: Mps1 (cat. no. ab11108; 1:150; Abcam), MAD1 (cat. no. ab175245; 1:100; Abcam), Bub1 (cat. no. ab54893; 1:200; Abcam), AKT (cat. no. 9272S; 1:200; Cell Signaling Technology, Inc.) and mTOR (cat. no. ab2732; 1:150; Abcam). Tissue sections were incubated with primary antibodies at 4°C overnight, and then incubated with horseradish peroxidase-labeled secondary antibody (1:200; cat. no. KXX0022; Dako; Agilent Technologies, Inc.) at room temperature for 1 h. 3′,3′-Diaminobenzidine was used as a chromogen and the slides were incubated for 5 min. The sections were counterstained in Meyer's hematoxylin, washed again and dehydrated with ethanol and xylene prior to mounting. For IHC, brown particle staining was determined in OS tissues and normal tissues as a measure of positive staining. Tissue sections were assessed using an Olympus BX-41 light microscope to obtain high-resolution images (magnification, ×200; Olympus Corporation). Aperio Cytoplasma 2 software (Leica Microsystems, Inc.) was used to evaluate the intensity of IHC staining as follows: i) 0–25 points, (−); ii) 25–50 points, (+); iii) 50–75 points, (++); and iv) >130 points, (+++).

#### Cell culture

As for the selection of cell lines for constructing OS model in nude mice, 143B and MG63 cells are widely used OS cells. Several relevant studies have shown that 143B and MG63 cells are mostly used in antitumor experiments for the occurrence and development of OS (31,32). The human OS cell lines, 143B and MG63, were purchased from ScienCell Laboratories. Cells were cultured in high-glucose Dulbecco Modified Eagle's Medium (DMEM; HyClone; Cytiva) supplemented with 10% fetal bovine serum (FBS; HyClone; Cytiva), and 100 U/ml penicillin/streptomycin (Gibco; Thermo Fisher Scientific, Inc.) Cells were maintained in a humidified atmosphere at 37°C with 5% CO_2_. MG63 and 143B OS cells treated with PTX were used for subcutaneous tumor-forming studies in nude mice. Briefly, cells were treated with 1 µmol/l PTX for 24 h then transfected with 1 µg short hairpin RNA (shRNA) plasmid targeting Mps1 or with 1 µg Mps1 overexpression plasmid. The shRNA plasmid was constructed using the GV248 vector (Shanghai GeneChem Co., Ltd.). The overexpression plasmid (Mps1 Genbank ID, NM_003318) was constructed using the GV358 vector (Shanghai GeneChem Co., Ltd.). Both plasmids were transfected into cells using Lipofectamine^®^ 2000 for 60 h (with the medium replaced every day) following the manufacturer's instructions (Invitrogen; Thermo Fisher Scientific, Inc.) and then maintained in a humidified atmosphere at 37°C with 5% CO_2_. Subsequent experiments were performed at 48 h post-transfection.

#### Nude mouse tumor model

BALB/c-nu nude mice (male, 5-weeks-old; n=36; provided by Hunan Changsha Tianqin Biotechnology Co., Ltd.) were purchased from and housed in specific pathogen-free conditions (temperature 20–26°C; relative humidity, 40–70%; 12-h light-dark cycle) at Youjiang Medical University for Nationalities. All animal experiments were approved by the institute's Animal Ethics Committee (approval no. 20200122). In an initial experiment, mice were injected with MG63 cells then mice divided into two groups (n=6/group): The control (saline group) or the PTX-treated group. In a separate experiment, mice were divided into four groups (n=6/group): i) Saline (control); ii) PTX-treated; iii) sh-MPS1-transfected and iv) PTX-treated and sh-Mps1-transfected. MG63 cells were harvested, counted, and resuspended in phosphate buffered saline (PBS) at a final concentration of 2×10^7^ cells/ml. Subsequently, 1×10^6^ cells in 50 µl PBS were injected into the armpits of mice using a 26-gauge needle. Animal health and behavior were monitored at the same time every day and tumor volumes were measured in living nude mice every week until week 5 (endpoint). No mice died during the experiment. The tumors were removed from the mice and weighed 5 weeks after the transplantation. Sodium pentobarbital (150 mg/kg; 1%) was injected into the mice, which were then sacrificed using cervical dislocation. The death of the mice was confirmed by the cessation of breathing and cardiac arrest. Tumor volumes were calculated using the formula: Volume=0.2618 × L × W × (L + W), where W and L represent average tumor width and length, respectively.

#### Flow cytometry analysis of cell apoptosis

After cell transfection and PTX treatment, cells were collected for apoptosis assays and were resuspended at 10^7^ cells/ml. A volume of 100 µl was added to a 5-ml flow tube, and apoptosis measured using an PE Annexin V Apoptosis Detection kit (BD Biosciences) according to manufacturer's instructions. Cells were incubated at room temperature and in the dark for 15 min. The data were acquired by flow cytometry using a FACScan (BD Biosciences) flow cytometer according to the manufacturer's instructions. FlowJo V10.0.7 (Tree Star, Inc.) was used for data analysis.

#### MTT assay

After digestion with 0.25% trypsin, 143B and MG63 cells were seeded in 96-well plates at 5×10^3^ cells/well, in DMEM containing 10% FBS, at a constant temperature of 37°C and 5% CO_2_. The MTT assay was performed at 0, 12, 24, 48 and 72 h. At the indicated timepoints, 20 µl MTT solution (5 mg/ml) was added to each well, and the plate placed into a 5% CO_2_ incubator at 37°C for 4 h. The supernatant was then discarded, 150 µl DMSO added to each well, and the plate incubated at room temperature for 10 min. Absorbance values were measured on an spectrophotometer at 490 nm. A growth curve was obtained by plotting the absorbance for each time point.

#### Scratch assay

The 143B and MG63 cells were seeded into 6-well plates at a density of 1×10^5^ cells/well, to assess migration. When cells covered 80–90% of the dish, a sterile 100-µl pipette tip was used to mark four scratches in each well. Cells were washed twice in PBS and cultured in serum-free medium, and images were taken at 0 and 24 h (magnification, ×4). Adobe Illustrator software (version CC 2019; Adobe Systems, Inc.) was used to measure the width of the scratches. Cell mobility=(0 h scratch width-48 h scratch width)/0 h scratch width ×100. The assay was performed three times.

#### Transwell migration assay

Transwell migration assays were performed using a Transwell chamber (Corning, Inc.). The cells were prepared into a suspension using the medium and then inoculated into a six-well plate at a rate of 1×10^4^ cells/well. Next, 150 µl suspended cells in serum-free medium were seeded into the upper Transwell chamber, and 500 µl DMEM medium supplemented with 20% FBS was added to the lower chamber. After 24 h, the cells were fixed with 4% paraformaldehyde at room temperature for >10 min and stained with 0.5% crystal violet at room temperature for 10 min. An inverted light microscope was used to examine the cells, and three random fields were observed to obtain average data.

#### Western blot analysis

Cells and tissues were lysed in 1X RIPA buffer (Beyotime Institute of Biotechnology) containing protease and phosphatase inhibitors. The protein extraction product was quantified by bicinchoninic acid assay (Beyotime Institute of Biotechnology). Proteins (50 µg) were loaded and separated on 10% SDS-polyacrylamide gels, then transferred to polyvinylidene difluoride membrane (Bio-Rad Laboratories, Inc.) using the wet transfer method. Each membrane was blocked with TBST (100 mM Tris-HCl, pH 7.5; 150 mM NaCl; 0.05% Tween-20) with 5% non-fat dried milk for 1 h at room temperature, then incubated with primary antibodies at 4°C overnight. The membranes were then further incubated with HRP-conjugated secondary antibodies (anti-rabbit IgG, cat. no. 7074, 1:2,000; or anti-mouse IgG, cat. no. 7076, 1:2,000; both Cell Signaling Technology, Inc.) for 1 h at room temperature. Protein bands were visualized with ECL kit (Amersham; Cytiva) and the protein expression levels were analyzed using ImageJ software (v1.48; National Institutes of Health). The following primary antibodies were used: i) Mps1 (Abcam; cat. no. ab11108; 1:1,000); ii) MAD1 (Abcam; cat. no. ab175245; 1:1,000); iii) Bub1 (Abcam; cat. no. ab54893; 1:1,000); iv) Ki67 (Abcam; cat. no. ab231172; 1:1,000); v) mTOR (Abcam; cat. no. ab2732; 1:2,000); vi) AKT (Cell Signaling Technology, Inc.; cat. no. 9272S; 1:1,000); and vii) β-actin (Abcam; cat. no. ab8226; 1:5,000).

#### Statistical analysis

Graph Pad Prism version 6.0 software (GraphPad Software, Inc.) was used for statistical analysis and plotting. The data are presented as the mean ± SD. Tukey's post hoc test was used following one-way ANOVA. Three replicates were carried out for cell experiments and six for animal experiments. P<0.05 was considered significantly different.

## Results

### 

#### Mps1 expression is upregulated in OS samples and associated with patient survival rate

**Figure 1.**
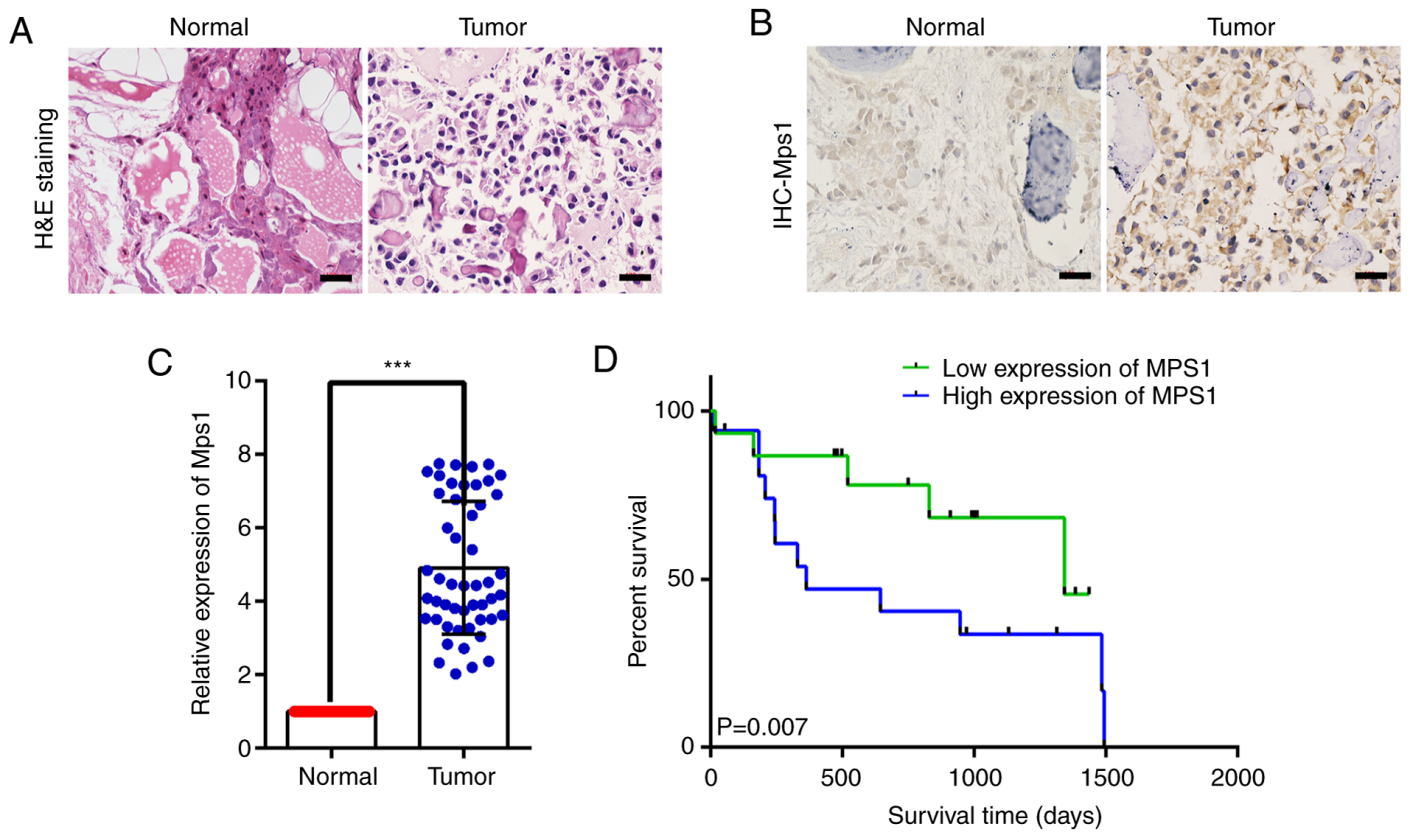
Mps1 expression is upregulated in OS samples and is associated with patient survival rate. (A) Representative images of H&E staining of tumor or normal adjacent tissue samples from patients with OS. Scale bar, 100 µm. (B) Representative images of Mps1 IHC staining in tumor or normal adjacent tissue samples from patients with OS. Scale bar, 100 µm. (C) Reverse transcription-quantitative PCR analysis of Mps1 expression levels in tumor or normal adjacent tissue samples from patients with OS. n=50 in each group. ***P<0.001, Welch's t-test. Data are normalized to GAPDH and presented as the mean ± SD. (D) Overall survival rate of patients with high or low Mps1 expression levels. OS, osteosarcoma; Mps1, monopolar spindle 1 kinase; H&E, hematoxylin and eosin; IHC, immunohistochemistry.

Mps1 expression in OS tissue was analyzed using HE staining and IHC. HE staining revealed that compared with normal tissue, OS tissue with obvious neoplastic osteoid tissue appeared in the background of malignant sarcoma cells, presenting typical neoplastic osteoid tissue, and OS tissue cells were full of distinctly heterogeneous neoplastic osteoblasts (Fig. 1A). IHC showed that Mps1 was localized to the cytoplasm and nuclei, although expression in OS tissue appeared higher than that in normal bone tissue (Fig. 1B).

Mps1 mRNA expression was analyzed using RT-qPCR in 50 clinical OS and adjacent normal tissue samples. Mps1 mRNA expression in OS tissue was significantly higher than that in adjacent normal bone tissue (Fig. 1C). In addition, a log-rank test was used to analyze Mps1 expression and survival rates of 50 clinical patients with OS. The overall survival rate of patients with high Mps1 expression was significantly lower than that of patients with low Mps1 expression (Fig. 1D). Taken together, these findings indicated that Mps1 expression was significantly upregulated in OS tissue and may be associated with overall survival of patients with OS.

#### Mps1 regulates SAC and Akt/mTOR signaling in OS

**Figure 2.**
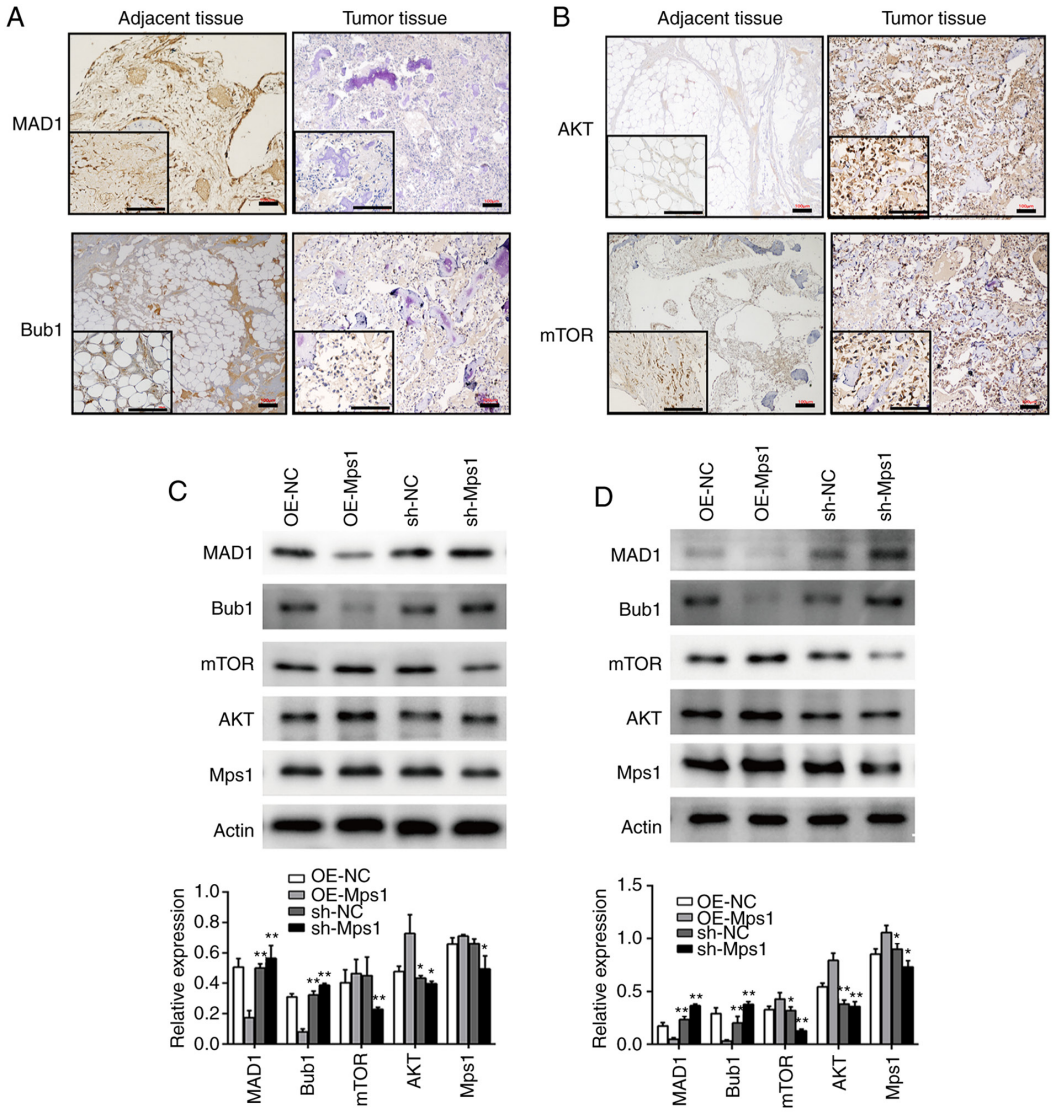
Mps1 regulates the spindle assembly checkpoint and Akt/mTOR signaling in osteosarcoma cells. (A and B) Representative images of (A) MAD1 and Bub1 or (B) AKT and mTOR immunohistochemical staining in tumor or normal adjacent tissue samples from patients with OS. Scale bar, 100 µm. (C and D) Western blot analysis with semi-quantified results of MAD1, Bub1, mTOR, AKT and Mps1 protein expression in (C) 143B or (D) MG63 cells transduced with OE-NC, OE-Mps1, sh-NC or sh-Mps1 lentivirus. *P<0.05, **P<0.01 vs. control. Mps1, monopolar spindle 1 kinase; OE, overexpression; sh, short hairpin RNA; NC, negative control.

To determine the role of Mps1 and PTX in modulating OS, SAC and Akt/mTOR signaling were examined. IHC of OS tissue revealed that expression levels of the SAC components, MAD1 and Bub1 in OS tissues were lower than in adjacent tissues (Fig. 2A). In contrast, Akt and mTOR expression levels in OS tissue were significantly higher than those of adjacent tissue samples (Fig. 2B). These results suggested that SAC may be impaired, whereas components of Akt/mTOR signaling were upregulated in OS tissue. Thus, it was hypothesized that SAC and the Akt-mTOR signaling pathway may play important roles in OS development. Next, the expression of SAC and Akt/mTOR signaling markers were analyzed following genetic manipulation of Mps1 expression *in vitro*. 143B and MG63 cells were transfected with an Mps1 overexpression vector (OE-Mps1) or shRNA-Mps1 lentiviral vector. Western blot analysis confirmed overexpression of Mps1 in the OE-Mps1 group and significantly decreased Mps1 expression in the shRNA-Mps1 group. Compared with the respective controls, MAD1 and Bub1 expression were downregulated and AKT and mTOR expression upregulated in the OE-Mps1 group, whereas the shRNA-Mps1 group showed the opposite results (Fig. 2C and D). Thus, Mps1 may regulate SAC and Akt/mTOR signaling in OS.

#### PTX inhibits Mps1 expression, impairs migration of OS cells and promotes cell apoptosis

**Figure 3.**
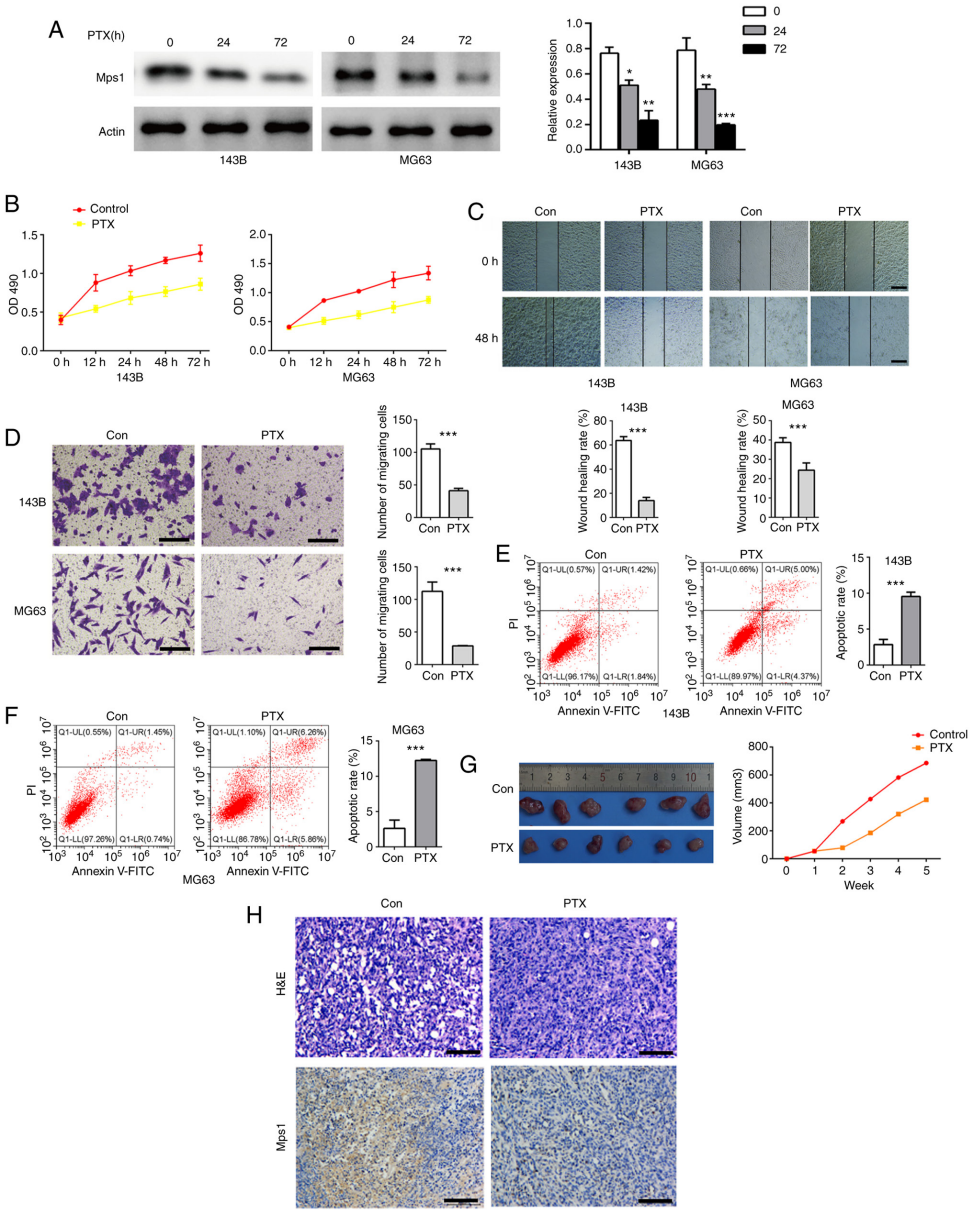
PTX inhibits Mps1 expression and migration of osteosarcoma cells and promotes their apoptosis. (A) Western blot analysis with semi-quantified results of Mps1 protein expression in 143B or MG63 cells after treatment with PTX for 0, 24 or 72 h. (B) Proliferation of 143B and MG63 cells after treatment with PTX for 0, 12, 24, 48 and 72 h. (C) Representative photomicrographs of wound healing assays in 143B and MG63 cells at 0 and 24 h (magnification, ×100). (D) Transwell assay results of 143B and MG63 cells treated with PTX for 24 h. (E and F) Flow cytometry analysis of apoptosis in (E) 143B and (F) MG63 cells treated with PTX for 24 h. (G) Representative images and quantification of the volume of the xenograft tumors in the control and PTX-treated groups. (H) H&E and immunohistochemical staining of Mps1 in tumors from the control and PTX-treated groups. Scale bar, 100 µm. *P<0.05, **P<0.01, ***P<0.001 vs. control. Data are presented as the mean ± SD. Mps1, monopolar spindle 1 kinase; PTX, paclitaxel; OD, optical density; H&E, hematoxylin and eosin.

To investigate the impact of PTX on Mps1 expression, the OS cell lines, 143B and MG63, were treated with PTX for 12, 24, 48 or 72 h. Western blot analysis showed that Mps1 expression significantly decreased in cells cultured with PTX for 24 and 72 h (Fig. 3A). PTX also inhibited OS cell viability in a time-dependent manner (Fig. 3B). From the scratch assay data, it was observed that cell migration was inhibited by PTX treatment in both 143B and MG63 cells (Fig. 3C). Transwell results showed that PTX inhibited cell migration, and flow cytometry data showed PTX induced cell apoptosis (Fig. 3D-F) compared with the control. Next, MG63 cells treated with 1 µmol/l PTX were used to construct a nude mouse OS model, and tumor volume growth was recorded in nude mice over 5 weeks. Tumor histology and Mps1 expression in OS tissue was analyzed using HE staining and IHC, respectively. Tumor volumes of PTX exposed nude mice were significantly smaller than those of the control group (Fig. 3G). HE staining revealed no significant differences in the morphology of MG63 tumor tissues, and IHC confirmed a significant decrease in Mps1 expression (Fig. 3H) compared with the control. In summary, these data suggested that PTX downregulated Mps1 expression both *in vitro* and *in vivo*, inhibited OS cell proliferation, migration and promoted OS cell apoptosis.

#### PTX combined with Mps1 knockdown exhibits stronger anti-tumor effects in OS

**Figure 4.**
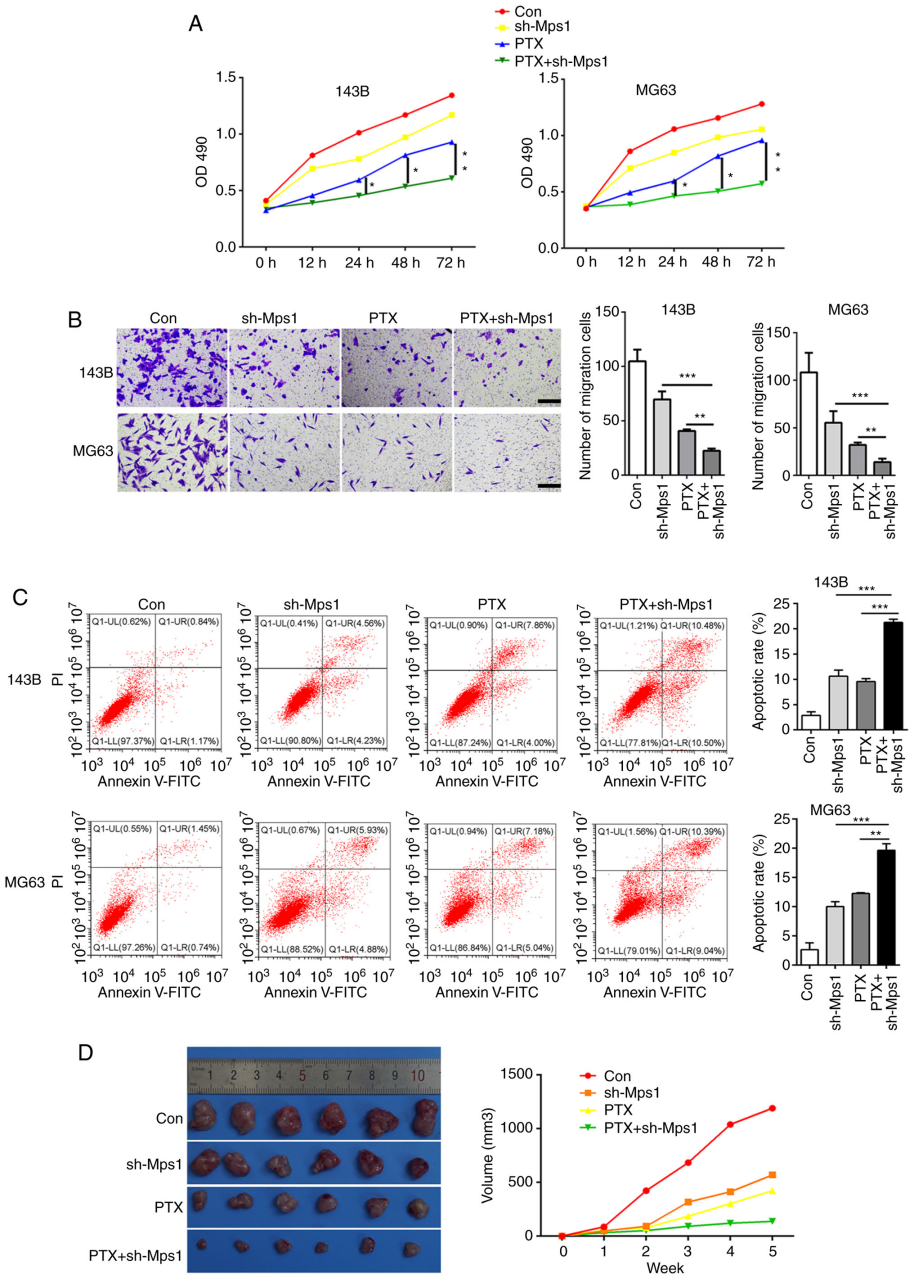
PTX combined with Mps1 knockdown exhibits strong anti-tumor effects in osteosarcoma cells. (A) Cell proliferation curve of 143B and MG63 cells in the con, sh-Mps1, PTX and PTX + sh-Mps1 groups. (B) Transwell assay results of 143B and MG63 cells in the con, sh-Mps1, PTX and PTX + sh-Mps1 for 24 h. (C) Flow cytometry analysis of apoptosis in 143B and MG63 cells in the con, sh-Mps1, PTX and PTX + sh-Mps1 groups. (D) Representative images and quantification of the volume of the xenograft tumors in the con, sh-Mps1, PTX and PTX + sh-Mps1 groups. *P<0.05, **P<0.01, ***P<0.001. Data are presented as the mean ± SD. Mps1, monopolar spindle 1 kinase; PTX, paclitaxel; sh, short hairpin RNA; OD, optical density; con, control.

To determine whether PTX in combination with Mps1 knockdown would have an improved effect against OS compared with PTX alone, the 143B and MG63 cell lines were transfected with the shRNA-Mps1 lentiviral vector and treated with PTX. The OS cells were divided into four groups: i) Control (saline); ii) shRNA-Mps1; iii) PTX alone; and iv) PTX + shRNA-Mps1. The MTT assay revealed that PTX + shRNA-Mps1 showed stronger cytotoxicity than PTX alone in OS cell, and this inhibitory effect increased with longer exposure times (Fig. 4A). Furthermore, cell migration was reduced, and the apoptosis rate was higher in PTX + shRNA-Mps1cells compared with the sh-Mps1 or PTX alone groups (Fig. 4B and C). The cells from different groups were then injected into nude mice. The tumor volume was smaller in mice in the PTX+ shRNA-Mps1 group compared with the shMps1 or PTX alone groups (Fig. 4D). Thus, these data indicated that PTX in combination with shRNA-Mps1 exerted a stronger antitumor effect on OS than shMps1 or PTX alone.

#### PTX in combination with Mps1 knockdown mediates SAC and Akt/mTOR signaling

**Figure 5.**
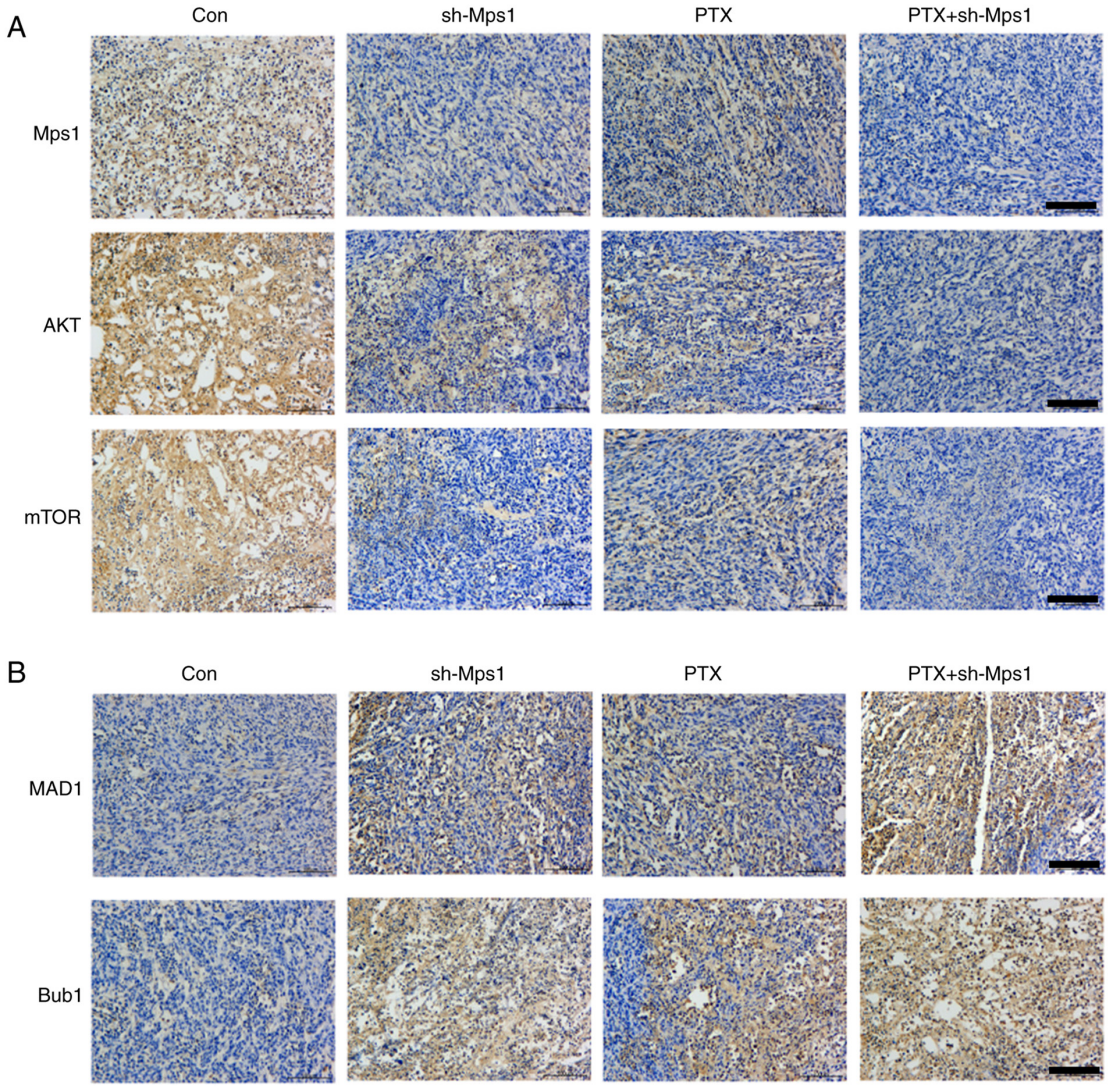
PTX in combination with Mps1 knockdown mediates spindle assembly checkpoint and Akt/mTOR signaling to inhibit osteosarcoma development. Representative image of (A) Mps1, AKT and mTOR or (B) MAD1 and Bub1 immunohistochemical staining in tumor tissues sample from the control, sh-Mps1, PTX and PTX + shMps-1 groups. Scale bar, 100 µm. Mps1, monopolar spindle 1 kinase; PTX, paclitaxel.

To examine the effects of PTX in combination with Mps1 knockdown on SAC and Akt/mTOR signaling in OS, key biomarkers were examined in all groups. IHC results suggested that Mps1, Akt and mTOR expression levels were markedly lower, whereas those of MAD1 and Bub1 were higher in the shRNA-Mps1 group compared with the control group. The data from the PTX alone group were comparable with shRNA-Mps1 alone. In addition, the PTX + shRNA-Mps1 groups showed the lowest Akt and mTOR expression levels and the highest MAD1 and Bub1 expression levels (Fig. 5). In conclusion, PTX affected SAC, as well as Akt and mTOR expression. In addition, PTX in combination with Mps1 knockdown exerted a stronger effect than either condition alone on SAC, and the Akt/mTOR pathways.

## Discussion

OS is caused by several factors, including aberrant differentiation of mesenchymal cells and tumor suppressor genes, oncogene activation, epigenetic events and cytokine production (33). Although OS therapeutics have improved after decades of research, long-term survival rates and prognosis for OS are still low due to early metastasis and high malignancy (2,34). These facts indicate the requirement for more mechanistic studies and the development of novel and more effective treatment strategies to elucidate disease pathogenesis, in order to improve patient survival times and prognosis. Currently, OS treatment strategies include neoadjuvant chemotherapies combined with surgical treatments and postoperative adjuvant chemotherapies (35). PTX is an effective anticancer drug that has been widely used to clinically combat several cancer types (36). PTX is currently used for the treatment of OS in preclinical research settings, and has shown promising clinical applications (37). In the present study, PTX exerted antitumor effects on OS, both *in vivo* and *in vitro*.

As a novel target and biomarker for cancer, Mps1 inhibitors effectively inhibit cancer cell proliferation and result in significantly improved survival (38). In the present study, high Mps1 expression was associated with poor patient survival. Mps1 was significantly upregulated in OS compared with normal adjacent tissues. Moreover, Mps1 modulation appeared to interfere with OS cell proliferation and development. Thus, to further investigate the anti-tumor effects of PTX in combination with Mps1 modulation in OS, several experiments were conducted, which showed that PTX combined with Mps1 inhibition exerted improved effects against OS compared with PTX alone. Mps1 is a bispecific protein kinase upstream of SAC that plays a key role in the precise separation of chromosomes during mitosis (13). In the present study, the downstream SAC markers, MAD1 and Bub1 were downregulated in OS tissue. Regulating Mps1 expression affected MAD1 and Bub1 expression, and was related to OS cell growth characteristics. Thus, the combination of PTX and Mps1 inhibition in OS cells appeared to upregulate SAC, and inhibit the oncogenic characteristics of OS cells. However, whether these mechanisms are directly or indirectly regulated by Mps1 to MAD1 and Bub1 has not yet been clearly established, and requires more research.

The Akt/mTOR pathway has been widely studied in various tumor cells, with its role in OS attracting considerable attention (23,24,39). In this study, Akt and mTOR expression levels were upregulated in OS tissue and affected by PTX treatment. Moreover, PTX downregulated the expression of Akt and mTOR to inhibit cancer-related OS characteristics. PTX combined with Mps1 knockdown significantly downregulated Akt and mTOR in OS cells.

The present study had some limitations. The occurrence and development of OS is not regulated by a single signaling pathway, but more likely regulated via multiple signaling pathways. The possible relationship between the Mps1 gene and other signaling pathways implicated in OS occurrence and development were not examined. The failure to establish a lung metastatic model of OS in nude mice is also a limitation of this study. Due to the limitation of research conditions in Guangxi province, OS samples are relatively rare, so it is difficult to draw reliable conclusions by distinguishing different subtypes of OS. In addition, the relationship between the Mps1 gene and the degree of differentiation, type and tumor size of OS tumors were not statistically analyzed.

In summary, the present findings showed that PTX had effective antitumor effects, and that PTX combined with Mps1 inhibition improved the anti-OS effects. Mps1 inhibition significantly enhanced the tumor inhibition effects of PTX. This treatment strategy not only prevented OS cell proliferation and development *in vitro*, but also inhibited the growth of OS transplanted tumors in nude mice. Additionally, PTX in combination with Mps1 inhibition upregulated MAD1 and Bub1 expression, potentially enhancing SAC in tumor cells. Moreover, the Akt/mTOR pathway may also represent a target pathway potentially regulated by PTX and Mps1, and that Akt/mTOR pathway downregulation could be of great significance in inhibiting OS development. Therefore, PTX in combination with Mps1 inhibition may have broad application prospects for molecular therapeutics against OS.

## Data Availability

The datasets used and/or analyzed during the current study are available from the corresponding author on reasonable request.
